# AST and ALT APRI Scores and Dysglycemia in Saudi Arabia: A Retrospective Population Study

**DOI:** 10.3390/life13091881

**Published:** 2023-09-07

**Authors:** Yazeed Alshuweishi, Mohammed Alfaifi, Yousef Almoghrabi, Mohammad A. Alfhili

**Affiliations:** 1 Chair of Medical and Molecular Genetics Research, Department of Clinical Laboratory Sciences, College of Applied Medical Sciences, King Saud University, Riyadh 12372, Saudi Arabia; yalshuweishi@ksu.edu.sa; 2 Department of Clinical Laboratory Sciences, College of Applied Medical Sciences, King Khalid University, Abha 61421, Saudi Arabia; mhalfaifi@kku.edu.sa; 3 Department of Clinical Biochemistry, Faculty of Medicine, King Abdulaziz University, Jeddah 21589, Saudi Arabia; yalmoghrabi@kau.edu.sa; 4 Department of Clinical Pathology, Al Borg Diagnostics, Jeddah 23226, Saudi Arabia

**Keywords:** hyperglycemia, liver scores, biomarker, Saudi Arabia

## Abstract

Background: Hyperglycemia is a common symptom of numerous conditions, most notably diabetes mellitus and Cushing’s syndrome, and the liver plays a pivotal role in the regulation of glucose metabolism. The AST–platelet ratio index (AST APRI score) and ALT–platelet ratio index (ALT APRI score) are novel parameters whose association with circulating glucose levels remains poorly studied. Methods: Laboratory data of 14,177 subjects were retrospectively analyzed for the association between AST and ALT APRI scores and fasting blood glucose (FBG) using the Mann–Whitney U and Kruskal–Wallis tests, Spearman’s rank correlation coefficient, prevalence and odds ratio (OR) and ROC curve analysis. Results: AST and ALT APRI scores showed progressive increases with FBG, and the mean FBG was significantly higher in subjects with high AST (104.9 ± 0.33 to 120.8 ± 3.27, *p* < 0.0001) and ALT (104.7 ± 0.34 to 111.6 ± 1.30, *p* < 0.0001) APRI scores. However, the AST APRI score but not the ALT APRI score was affected by age and gender. Notably, both elevated AST and ALT APRI scores were more prevalent in hyperglycemic subjects irrespective of gender and were associated with FBG, albeit through mediator variables. Increased AST (OR = 2.55, 95% CI: 1.46–2.06, *p* < 0.0001) and ALT (OR = 1.73, 95% CI: 1.46–2.06, *p* < 0.0001) APRI scores carried a significantly higher risk for hyperglycemia. Importantly, the ALT APRI score was superior to that of the AST APRI score in distinguishing hyperglycemic subjects. Conclusions: The AST and ALT APRI scores are inexpensive, novel markers of FBG and may serve as supportive evidence in the diagnosis and management of hyperglycemic conditions.

## 1. Introduction

Type 2 diabetes (T2D) is characterized as a multifactorial disorder that can be generated secondary to the presence of a genetic predisposition that is triggered by environmental factors [1]. The pathophysiology of T2D is complex and is associated with irreversible risk factors, such as age, genetics and race, and reversible factors, such as diet, physical activity and smoking [2]. According to the International Diabetes Federation (IDF), 17.7% of the Saudi adult population suffers from T2D, which is the second highest prevalence in the region and seventh worldwide [3]. T2D has been related to various liver illnesses, such as NAFLD, hepatocellular carcinoma and cirrhosis [4,5,6].

The liver has an essential part in the maintenance of glycemic homeostasis by balancing the uptake and storage of glucose via glycogenesis and the release of glucose via glycogenolysis and gluconeogenesis [7,8]. The health of the liver is closely tied to blood glucose regulation and insulin resistance. The liver enzymes alanine aminotransferase (ALT) and aspartate aminotransferase (AST) are central to the gluconeogenesis process in which glucose is synthesized from the amino acids alanine and aspartate and then exported to the blood circulation [9]. ALT is highly expressed in hepatocytes, and thus, abnormal levels of ALT tend to be more specific to liver injury [10]. Moreover, AST is found in several tissues, including the liver, muscle, the brain, the kidneys and the lungs [10]. Elevated levels of ALT and AST in the bloodstream are generally considered a sign of liver tissue damage [10]. Given the role of the liver in glucose metabolism, it is not surprising that several markers of liver injury, including AST, ALT, alkaline phosphatase (ALP) and γ-glutamyl-transferase (GGT), have been associated with insulin resistance and the risk of diabetes [11,12]. Additionally, there is an increased risk of developing T2D in patients with NAFLD, while there is a higher occurrence of steatohepatitis and liver fibrosis in patients with T2D [4,13]. This shows a complex relationship between liver disease and diabetes where one contributes to the progression of the other. Based on these observations, specific focus has been made on the contribution of liver function tests to the prediction of dysglycemia and T2D development. In this respect, multiple studies showed a significant relationship between liver enzymes and T2D in Asian [12,14], European [15,16] and American populations [17,18]. For instance, Kown et al. demonstrated that a high ALT/AST ratio was significantly correlated with impaired fasting glucose and HOMA-IR in 16,371 Korean adults [19]. Concerning the Saudi population, Alzahrani et al. similarly showed that higher levels of AST and ALT were detected in diabetic patients [20]. Based on these studies, the use of liver function parameters as a predictive model for the risk of developing T2D is an important strategy to identify the population at high risk of developing T2D.

The AST-to-platelet ratio index (APRI) is one of the serum biomarker indexes that were originally developed to predict severe liver fibrosis with a high diagnostic performance [21]. Particularly, this index has the advantage of including only two common and inexpensive markers: namely, AST and platelet count laboratory tests. The APRI score has been shown to possess a predictive value for conditions such as HELLP (hemolysis, elevated liver enzyme levels, low platelet counts) syndrome and cholestasis [22,23]. While others have used the GTT-to-platelet ratio (GPR) or ALP-to-platelet ratio (APPRI) as markers to predict liver fibrosis and cirrhosis in chronic hepatitis B and individuals with NAFLD [24,25], far less studies have tested the clinical utility of ALT instead of AST in calculating the APRI score index.

Considering the high prevalence of diabetes in Saudi Arabia and its implications on liver diseases, it is of importance to improve screening and early detection efforts. Although the relevance of abnormal liver profiles in the setting of diabetes is well documented, there is a paucity of studies that used a population-based approach to examine the relationship between the APRI score and changes in the concentration of fasting blood glucose (FBG). Thus, the current study aimed to examine the association of the APRI score and glycemic status in a large Saudi population with a greater emphasis on evaluating the impact of sex and age in the association estimate, and the diagnostic accuracy of the APRI score for blood glucose level. Additionally, we sought to compare the clinical utility between the AST–platelet ratio score index (AST APRI score) and ALT–platelet ratio index (ALT APRI score) in light of dysglycemia.

## 2. Materials and Methods

### 2.1. Study Design and Data Collection

The study protocol was approved by the Biomedical Ethics Unit of Al Borg Diagnostics (approval code is #07/21, approved by Al Borg Diagnostics on 27 December 2021). Subject consent was waived as the study was retrospective. Age, gender and laboratory data for 14,177 subjects whose results included both fasting blood glucose (FBG) and platelet counts, as well as liver enzymes AST and ALT were collected from 2014 to 2019. Males and females were separated, and age groups were categorized as follows: young (<18 years), young adults (18–39 years), adults (40–64 years) and elderlies (≥65 years), as previously reported [26]. Normoglycemia (NG) was set at an FBG of <100 mg/dL, impaired fasting glycemia (IFG) at an FBG between 100 and 125 mg/dL and hyperglycemia (HG) at an FBG of ≥126 mg/dL in accordance with the ADA guidelines [27]. The AST APRI score was calculated as {(AST/upper limit of normal AST) × 100/platelet count} [28]. Similarly, the ALT APRI score was calculated as {(ALT/upper limit of normal ALT) × 100/platelet count}. The upper limits of normal ALT and AST used in this study were 40 U/L, and an APRI score of ≥0.5 was considered high in accordance with previous studies [28,29].

### 2.2. Statistics

Data are shown as the means ± 95% CI or SEM as indicated. The means of two groups were compared with the Mann–Whitney U test and of three groups with Kruskal–Wallis one-way analysis of variance followed by Dunn’s multiple comparisons test. Associations were assessed with simple linear regression analysis and by calculations of the prevalence risk (PR) and odds ratio (OR). Sensitivity and specificity were examined with ROC curve analysis and area under the curve (AUC) determination. GraphPad Prism v9.2.0 (GraphPad Software, Inc., San Diego, CA, USA) was used for the statistical analysis, and a *p* value of <0.05 was considered statistically significant.

## 3. Results

### 3.1. Baseline Characteristics

As shown in Table 1, 14,177 subjects were included in this study of which 5928 were male (41.81%), 8210 were female (57.91%), and 39 were unknown (0.28%). The subjects were divided based on the concentration of FBG in which 9079 subjects were normoglycemic, 3310 subjects had impaired fasting glycemia, and 1788 subjects were hyperglycemic. The baseline characteristics of the study subjects are shown in Table 2. The mean age of the subjects in the NG, IFG and HG groups was 41.14 (±0.16), 41.19 (±0.28) and 43.15 (±0.41) years, respectively. The mean age of the IFG and HG groups was slightly yet significantly higher than that in the NG group. The mean platelet absolute count in the NG, IFG and HG groups was 281.9 (±0.78), 270.8 (±1.27) and 259.8 (±1.77), respectively. There was a significant reduction in the level of the platelet count in the IFG and HG groups compared to that in the NG group. For the liver enzymes, there was a significant increase in the level of AST and ALT enzymes in the IFG and HG groups compared to that in the NG group. The mean of AST in the NG, IFG and HG groups was 20.31 (±0.13), 21.3 (±0.20) and 21.01 (±0.29), respectively. The mean of ALT in the NG, IFG and HG groups was 22.18 (±0.19), 26.25 (±0.37) and 27.27 (±0.50), respectively.

### 3.2. Levels of APRI Scores Were Elevated in the IFG and HG Groups

**Figure 1:**
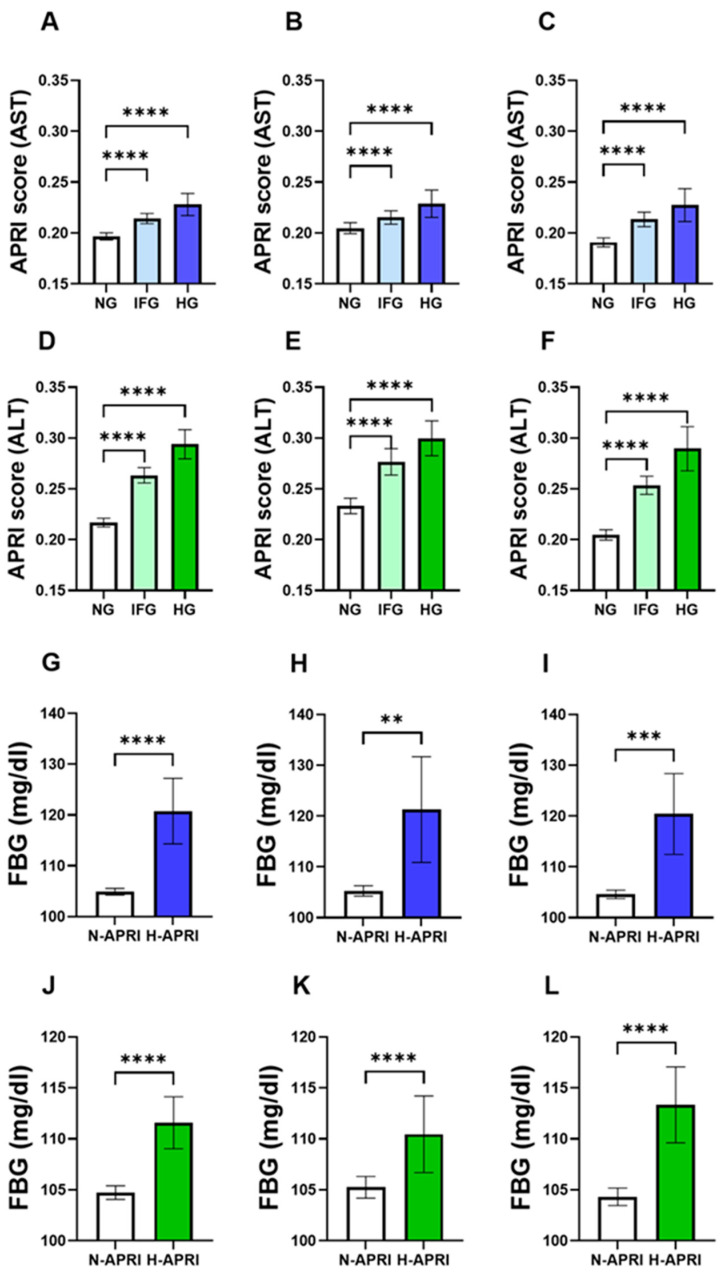
Changes in the APRI score levels in light of FBG. Means ± 95% CI of the AST APRI score of subjects with NG, IFG and HG in (**A**) both genders, (**B**) males and (**C**) females. Means ± 95% CI of the ALT APRI score of subjects with NG, IFG and HG in (**D**) both genders, (**E**) males and (**F**) females. Means ± 95% CI of FBG concentrations in the normal AST APRI score (N-APRI) and high AST APRI score (H-APRI) groups in (**G**) both genders, (**H**) males and (**I**) females. Means ± 95% CI of FBG concentrations in the normal ALT APRI score (N-APRI) and high ALT APRI score (H-APRI) groups in (**J**) both genders, (**K**) males and (**L**) females. ** *p* < 0.01, *** *p* < 0.001 and **** *p* < 0.0001.

When the APRI score index was calculated based on the AST levels, we observed a significant increase in the level of the APRI score in the IFG (0.214 ± 0.002) and HG (0.228 ± 0.006) groups in comparison to that in the NG group (0.197 ± 0.002) (Figure 1A). This was also true when males and females were considered alone as shown in Figure 1B,C. Additionally, the level of the APRI score calculated using ALT showed a profound increase in the IFG (0.263 ± 0.004) and HG (0.294 ± 0.007) groups in comparison to that in the NG group (0.217 ± 0.002) (Figure 1D). Similar patterns were observed when both genders were analyzed alone (Figure 1E,F).

### 3.3. FBG Is Significantly Elevated in Subjects with High APRI Score

To assess FBG concentration in light of the APRI score index of both enzymes, AST and ALT, subjects of both genders and across all age groups were stratified as having either a normal APRI score (N-APRI) or high APRI score (H-APRI). As shown in Figure 1G–I, the concentration of FBG was significantly elevated from 104.9 ± 0.3305 in the N-AST APRI group to 120.8 ± 3.274 in the H-AST APRI group. Similarly, the levels of FBG were increased in the H-ALT APRI score index to 111.6 ± 1.301 (Figure 1J–L). Similar patterns were observed when both genders were analyzed alone.

### 3.4. AST but Not ALT APRI Score Follows Distinct Age- and Gender-Specific Patterns

**Figure 2:**
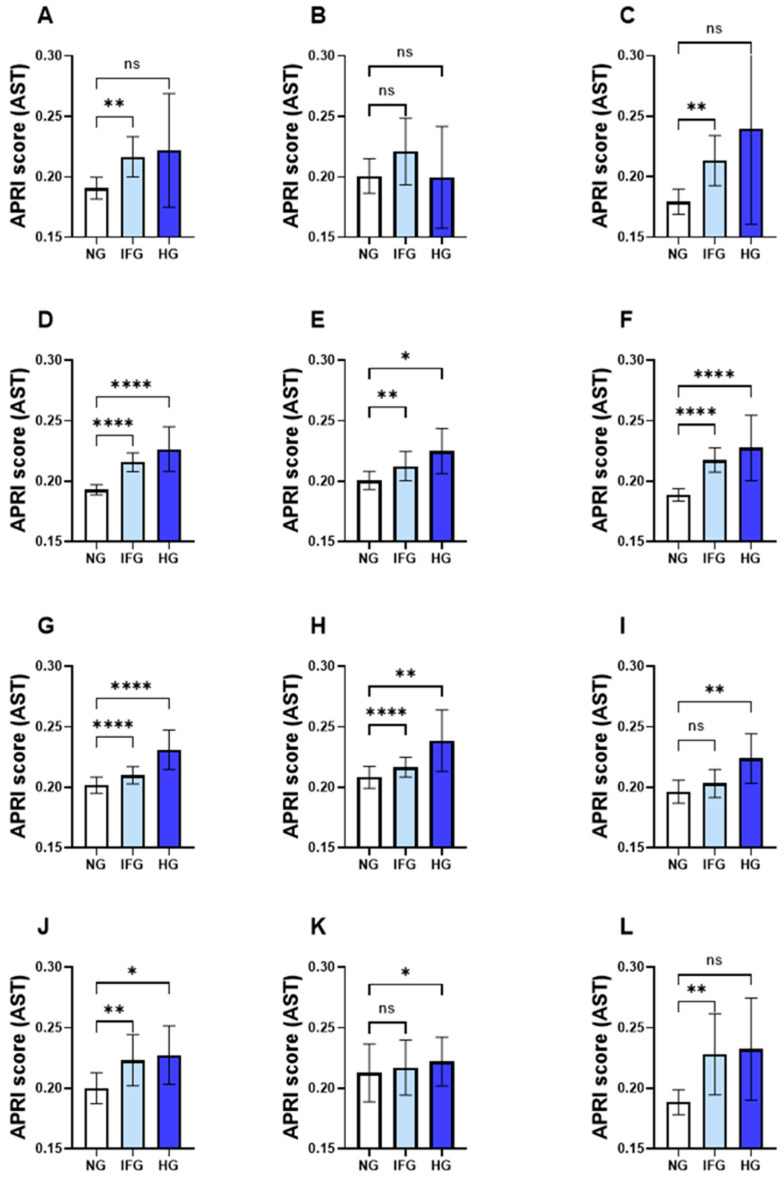
Impact of gender and age on the AST APRI score in light of FBG. Means ± 95% CI of the AST APRI score of subjects with NG, IFG and HG. Young groups are presented in (**A**) both genders, (**B**) males and (**C**) females. Young adults are presented in (**D**) both genders, (**E**) males and (**F**) females. Adults are presented in (**G**) both genders, (**H**) males and (**I**) females. Elderlies are presented in (**J**) both genders, (**K**) males and (**L**) females. ns indicates not significant while * *p* < 0.05, ** *p* < 0.01 and **** *p* < 0.0001.

**Figure 3:**
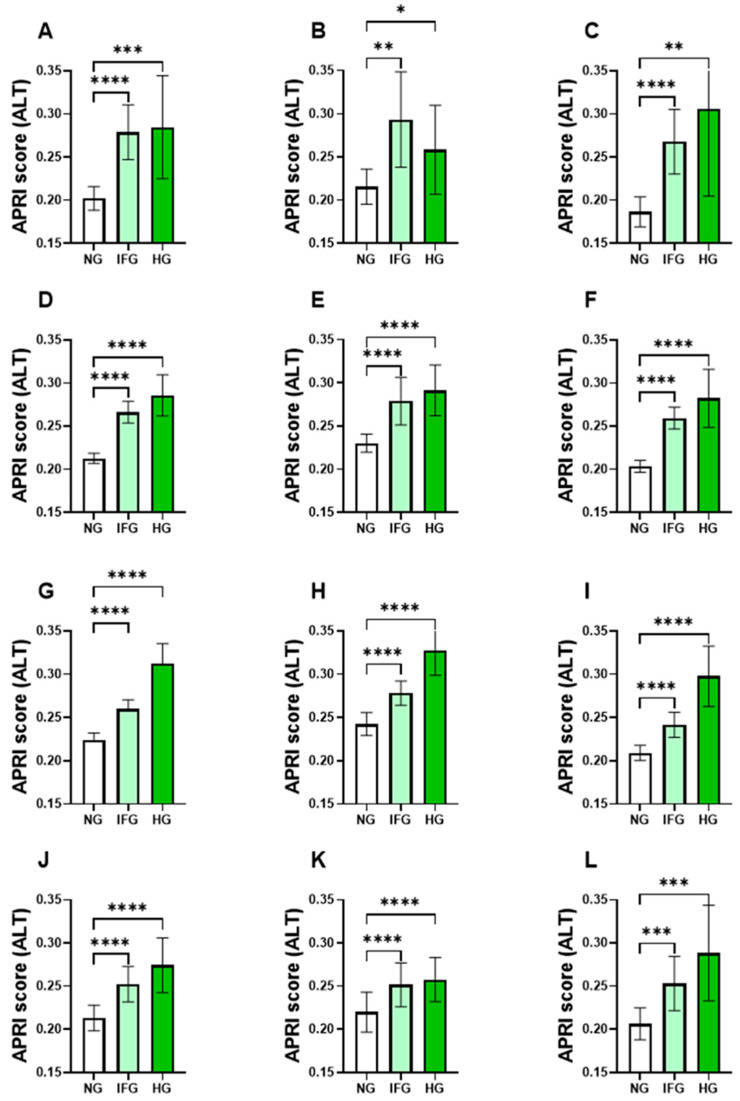
Impact of gender and age on the ALT APRI score in light of FBG. Means ± 95% CI of the ALT APRI score of subjects with NG, IFG and HG. Young groups are presented in (**A**) both genders, (**B**) males and (**C**) females. Young adults are presented in (**D**) both genders, (**E**) males and (**F**) females. Adults are presented in (**G**) both genders, (**H**) males and (**I**) females. Elderlies are presented in (**J**) both genders, (**K**) males and (**L**) females. * *p* < 0.05, ** *p* < 0.01, *** *p* < 0.001 and **** *p* < 0.0001.

As shown in Figure 2, the AST APRI score index was not able to distinguish between NG and HG in the male and female young groups (Figure 2B,C). Moreover, adult females had a similar level of AST APRI score between NG and IFG (Figure 2H,I). Moreover, female but not male elderlies failed to discriminate between the NG and HG groups (Figure 2K,L). On the other hand, the ALT APRI score across all age groups and in both IFG and HG subjects was elevated, as shown in the young group (Figure 3A–C), young adult group (Figure 3D–F), adult group (Figure 3G–I) and elderlies (Figure 3J–L) compared to that in their NG counterparts.

### 3.5. Prevalence of Hyperglycemia in High APRI Score

As shown in Table 3, the prevalence of normal AST APRI scores in the NG subjects in the studied population was extremely high, totaling 98.24% in both genders, 97.88% in males and 98.51% in females. These percentages were reduced in the HG subjects to 95.64% in both genders, 95.16% in males and 95.96% in females. In a comparison between NG and HG, the prevalence of high AST APRI scores increased from 1.76% to 4.36% in both genders, 2.12% to 4.84% in males and 1.49% to 4.04% in females. Moreover, a normal ALT APRI score from both genders was prevalent in 93.75% in the NG group, while it was less prevalent in the HG group (89.65%). Gender-specific analysis showed that a normal ALT APRI score was prevalent in 92.39% in males and 94.77% in females of the NG group, and these were reduced to 88.38% in males and 90.65 in females from the HG group. In a comparison between NG and HG, high ALT APRI scores increased from 6.25% to 10.35% in both genders, 7.61% to 11.52% in males and 5.23% to 9.35% in females.

### 3.6. Association of APRI Scores with FBG Concentration

**Figure 4:**
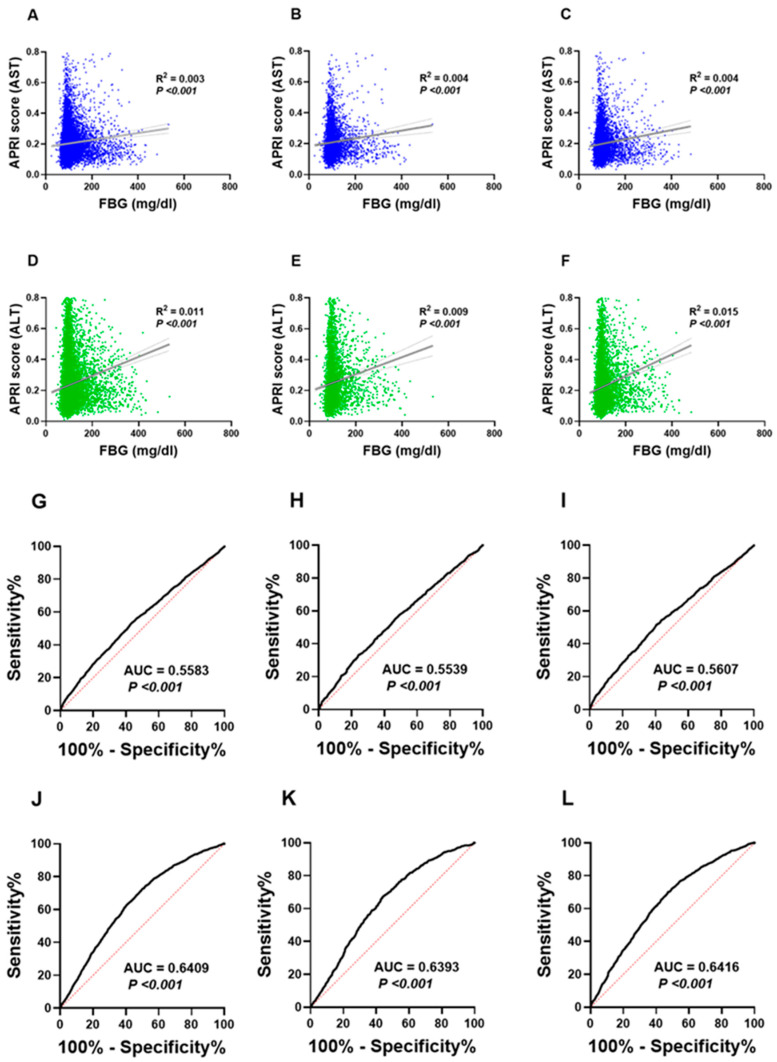
Association of the APRI score and FBG. Simple linear regression of the association between the AST APRI score and FBG concentration (**A**) in both genders, (**B**) in males and (**C**) in females. Simple linear regression of the association between the ALT APRI score and FBG concentration (**D**) in both genders, (**E**) in males and (**F**) in females. ROC curves of the AST APRI score and FBG concentration (**G**) in both genders, (**H**) in males and (**I**) in females. ROC curves of the ALT APRI score and FBG concentration (**J**) in both genders, (**K**) in males and (**L**) in females.

As depicted in Figure 4A, simple linear regression analysis revealed a significant yet weak association between the AST APRI score and FBG concentrations (R^2^ = 0.003 and *p* > 0.001). Similar patterns were observed between the AST APRI score and FBG when males and females were analyzed separately (Figure 4B,C). Furthermore, the ALT APRI score obtained better correlation with the FBG values (R^2^ = 0.01 and *p* > 0.001; Figure 4D). For the gender-specific analysis, the correlation between the ALT APRI score and FBG followed a similar pattern, although it was slightly stronger in females (Figure 4E,F).

To further assess the clinical utility of the APRI score in distinguishing subjects with NG from those with IFG and HG, we analyzed a receiver operating characteristic (ROC) curve for both genders and in males and females. As shown in Figure 4J, the area under the curve (AUC) generated with the ALT APRI score was 0.641 (*p* < 0.001), which is higher than the AUC achieved with the AST APRI score (0.558, *p* < 0.001; Figure 4G). The APRI score from either liver enzyme demonstrated that males and females exhibited a similar pattern when analyzed alone (Figure 4H,I,K,L).

### 3.7. Risk Assessment of APRI Score in Light of Hyperglycemia

The overall and gender-specific risk assessment analyses (Table 4) showed that the H-AST APRI score was associated with an increased risk of HG in both genders (RR = 2.48, 95% CI: 1.90–3.23, *p* < 0.0001), in males (RR = 2.28, 95% CI: 1.56–3.33, *p* < 0.0001) and in females (RR = 2.71, 95% CI: 1.87–3.92, *p* < 0.0001). Additionally, the H-AST APRI score was 2.55, 2.35 and 2.78 times more likely to have hyperglycemia in both genders, males and females, respectively. On the other side, the absolute risk of being hyperglycemic in the high ALT APRI group was 1.66, 1.51 and 1.79 in both genders, males and females, respectively. Subjects with high ALT APRI scores were 1.73, 1.58 and 1.87 times more likely to be hyperglycemic in both genders, males and females, respectively.

## 4. Discussion

Complete blood count and renal and liver function tests are routine, readily available, automated and inexpensive tests. The use of these tests in early diagnosis, prediction or prognostic monitoring of disease is of great importance to healthcare providers due to their quick access and ease of use. In this study, we retrospectively evaluated the association and diagnostic accuracy of the APRI score in the context of dysglycemia in the Saudi population. The main finding is that the APRI score significantly elevated in response to impaired fasting glucose (IFG) as well as hyperglycemia (HG). Additionally, this study presented a comparative analysis between the AST APRI score and ALT APRI score in which the ALT APRI score was found to be superior to the AST APRI score in predicting hyperglycemia as revealed with the ROC curve analysis. More importantly, the ALT APRI score is not associated with gender or age differences as demonstrated in our age- and gender-specific analyses. To the best of our knowledge, our study may be the first of its kind to investigate the association between the APRI score and dysglycemia in Saudi Arabia using a population-based approach.

Stratification of subjects based on FBG concentrations permitted the APRI score to distinguish IFG and HG from NG. In particular, the values of the AST APRI score were increased by approximately 8% and 15% in IFG and HG, respectively, compared to those of the NG control group, while the ALT APRI score values were elevated by 22% and 36% in IFG and HG, respectively, compared to those of the NG control group. The ability of the APRI score to discriminate IFG from NG suggests that the APRI score may be a sensitive marker of glucose disturbances. In a population of 1225 subjects, Matteis et al. demonstrated that the APRI score is elevated in prediabetics and significantly correlated with cardiovascular risk in non-metabolic and metabolic subjects [28]. Another report showed that the APRI score was high among those with worse glycemia, and a high APRI score was able to predict the development of diabetes [30].

Our study indicates that APRI scores are significantly yet weakly correlated with impaired fasting glucose levels. Similarly, a weak correlation between fasting glycemia and APRI score was observed in chronic hepatitis C patients [31]. This might indicate that the relationship is less likely to be direct with other intermediate variables involved. Given that T2D as well as prediabetic states are inflammatory conditions that are known to contribute to increased levels of reactive oxygen species [32], inflammation might be the linking factor between hyperglycemia and elevated liver enzymes. Indeed, a cross-sectional study in youth subjects detected a statistically significant relationship between increased BMI and insulin resistance in association with elevated liver enzymes, and this association was partially attributed to inflammation [33]. Additionally, persistent hyperglycemia and insulin resistance are the driving factors of intrahepatic lipid deposition that results in liver dysfunction [34]. Nevertheless, large and longitudinal studies are needed to further understand the crosstalk between liver inflammation, dysglycemia in T2D susceptibility and development.

One of the main findings in this study is the ability of the ALT APRI score to perform better than the AST APRI score in detecting glucose disturbances. This might be attributed to the individual role of both enzymes in liver glucose metabolism. A meta-analysis study that focused on AST and ALT levels detected an association between an increased risk of T2D and ALT but not AST [35]. Furthermore, ALT was positively correlated with FBG in a large Chinese population study [36]. The association between ALT levels and incident diabetes risk could be explained with several mechanisms. ALT is a specific marker of the accumulation of liver fat and plays a vital role in liver insulin sensitivity [37,38]. It was demonstrated that ALT levels are independently associated with hepatic insulin resistance in subjects with impaired fasting glucose [39].

Similarly, a negative correlation has been shown between ALT and insulin sensitivity using the euglycemic hyperinsulinemic clamp [40], which may contribute to the development of diabetes. On the other hand, data regarding the association between serum AST levels and incident type 2 diabetes risk were inconsistent [41,42]. In the present study, a poor diagnostic accuracy was observed with the AST APRI score in predicting hyperglycemia despite the ability of the AST APRI score to distinguish HG from NG. A prospective study among a middle-aged and elderly Chinese population showed that elevated serum ALT or AST levels were positively associated with an increased incident T2D risk with ALT levels more strongly associated with incident diabetes risk than AST levels [43]. A possible explanation could be that serum AST is a less specific marker of liver function because AST is also present in other tissues [44].

Interestingly, when stratified by both age and gender, the ALT APRI score was able to identify IFG and HG from NG in males and females and across all age groups. On the other hand, the AST APRI score failed to discriminate between NG and HG in young adults, and between NG and IFG in males but not females. Moreover, adult females had a similar level of AST APRI score between NG and IFG. In the elderly group, males and females exhibited opposite patterns where the AST APRI score was only sensitive to identify HG compared to NG in males and IFG compared to NG in females. These observations are not unprecedented since gender disparity is well documented in the predisposition, prevalence and disease progression of diabetes [45]. This seems to be related to the fact that sex hormones have a great impact on energy metabolism, body composition, vascular function and inflammatory responses [46]. It was reported that women usually show less tendency to develop fatty liver disease than men, and this protection was attributed partly to estrogen levels [47]. Also, women have higher levels of leptin and adiponectin, which are important adipokines in regulating food intake and energy expenditure and can also contribute to the development of peripheral insulin resistance [48]. In the context of insulin sensitivity, PTEN is a dual protein and lipid phosphatase that negatively regulates the insulin signaling pathway, and polymorphisms of the PTEN gene that lead to higher PTEN expression levels have been noted in diabetes [49,50]. Samaan and co-workers have demonstrated that women have lower muscle PTEN gene expression when compared to men, and this was coupled with an increased inactivation of the PTEN protein [51].

The strength of the current study is based on the large sample size, which is representative of the Saudi population, and automated data acquisition, which reduced analytical variability. However, given the cross-sectional nature of this study, it was not possible to determine a causal relationship between the APRI score and disturbances in glucose regulation. In addition, data regarding anthropometric variables, lifestyle habits, BMI, comorbidities, dietary intake and medication use were missing.

## 5. Conclusions

Altogether, this work shows, for the first time, that the AST and ALT APRI scores are associated with FBG in Saudi subjects, which argues for their further evaluation in longitudinal studies as screening, diagnostic and prognostic markers of glucose homeostasis. Elucidation of the underlying molecular mechanisms accounting for this association, such as the HOMA-IR index, is also warranted to pave the way to develop novel prophylactic and therapeutic interventions.

## Figures and Tables

**Table 1:** Gender distribution of the study subjects.

Gender	No. of Subjects (%)
Male	5928 (41.81)
Young	432 (3.05)
Young adults	2358 (16.63)
Adults	2580 (18.20)
Elderlies	558 (3.94)
Female	8210 (57.91)
Young	427 (3.01)
Young adults	4304 (30.36)
Adults	2855 (20.14)
Elderlies	624 (4.40)
Unknown	39 (0.28)

**Table 2:** Baseline characteristics of the study subjects.

Characteristic	NG (*n* = 9079)	IFG (*n* = 3310)	HG (*n* = 1788)	*p* Value
Age (years)	40.14 (±0.16)	41.19 (±0.28)	43.15 (±0.41)	<0.0001
Male (%)	41.44	42.36	42.73	
Female (%)	58.31	57.40	56.82	
WBC count (×10^6^/μL)	5.91 (±0.02)	6.03 (±0.03)	6.61 (±0.05)	<0.0001
RBC count (×10^6^/μL)	5.24 (±0.01)	5.37 (±0.01)	5.35 (±0.01)	<0.0001
Hemoglobin (g/dL)	14.06 (±0.02)	14.51 (±0.03)	14.64 (±0.05)	<0.0001
Platelet Count (×10^6^/mL)	281.9 (±0.78)	270.8 (±1.27)	259.8 (±1.77)	<0.0001
AST (U/L)	20.31 (±0.13)	21.3 (±0.20)	21.01 (±0.29)	<0.0001
ALT (U/L)	22.18 (±0.19)	26.25 (±0.37)	27.27 (±0.50)	<0.0001
Total Bilirubin	0.66 (±0.01)	0.63 (±0.01)	0.58 (±0.01)	<0.0001
Albumin (g/dL)	4.23 (±0.01)	4.20 (±0.01)	4.12 (±0.01)	<0.0001
Creatinine (mg/dL)	0.75 (±0.01)	0.81 (±0.01)	0.84 (±0.01)	<0.0001

**Table 3:** Prevalence of normoglycemia (NG) and hyperglycemia (HG) relative to the APRI score.

Parameter	NG	HG
Both		
N-APRI score (AST)	98.24	95.64
H-APRI score (AST)	1.76	4.36
N-APRI score (ALT)	93.75	89.65
H-APRI score (ALT)	6.25	10.35
Male		
N-APRI score (AST)	97.88	95.16
H-APRI score (AST)	2.12	4.84
N-APRI score (ALT)	92.39	88.48
H-APRI score (ALT)	7.61	11.52
Female		
N-APRI score (AST)	98.51	95.96
H-APRI score (AST)	1.49	4.04
N-APRI score (ALT)	94.77	90.65
H-APRI score (ALT)	5.23	9.35

**Table 4:** Risk assessment of elevated APRI scores and hyperglycemia.

		Score	95% CI	z Statistic	Significance Level
APRI score (AST)	PR				
	Both	2.48	1.90–3.23	6.69	*p* < 0.0001
	Male	2.28	1.56–3.33	4.24	*p* < 0.0001
	Female	2.71	1.87–3.92	5.26	*p* < 0.0001
	OR				
	Both	2.55	1.93–3.35	6.65	*p* < 0.0001
	Male	2.35	1.58–3.49	4.21	*p* < 0.0001
	Female	2.78	1.89–4.08	5.23	*p* < 0.0001
APRI score (ALT)	PR				
	Both	1.66	1.41–1.94	6.26	*p* < 0.0001
	Male	1.51	1.21–1.90	3.59	*p* = 0.0003
	Female	1.79	1.43–2.24	5.11	*p* < 0.0001
	OR				
	Both	1.73	1.46–2.06	6.18	*p* < 0.0001
	Male	1.58	1.23–2.03	3.55	*p* = 0.0004
	Female	1.87	1.47–2.39	5.04	*p* < 0.0001

## Data Availability

Data are available from the corresponding author upon reasonable request, and with permission of Al Borg Diagnostics.
